# Expression of Obesity Markers and Persistent Organic Pollutants Levels in Adipose Tissue of Obese Patients: Reinforcing the Obesogen Hypothesis?

**DOI:** 10.1371/journal.pone.0084816

**Published:** 2014-01-10

**Authors:** Anna Pereira-Fernandes, Eveline Dirinck, Alin C. Dirtu, Govindan Malarvannan, Adrian Covaci, Luc Van Gaal, Caroline Vanparys, Philippe G. Jorens, Ronny Blust

**Affiliations:** 1 Systemic Physiological and Ecotoxicological Research (SPHERE), Department of Biology, University of Antwerp, Antwerp, Belgium; 2 Department of Endocrinology, Diabetology and Metabolism, Antwerp University Hospital, University of Antwerp, Edegem, Belgium; 3 Toxicological Centre, University of Antwerp, Antwerp, Belgium; 4 Department of Clinical Pharmacology, Antwerp University Hospital, University of Antwerp, Edegem, Belgium; University of Texas, MD Anderson Cancer Center, United States of America

## Abstract

**Introduction:**

Persistent Organic Pollutants (POPs) accumulate in adipose tissue and some are described to possess endocrine disrupting capacities. Therefore, it is important to evaluate their effects on key endocrine pathways in adipose tissue (AT), to further evaluate their potential role in metabolic pathologies such as obesity.

**Objectives:**

The aim is twofold: (i) evaluate gene expression levels of obesity marker genes, i.e. the adipokines leptin (*LEP*), adiponectin (*ADIPOQ*) and Tumor Necrosis Factor α (*TNFα*) and the nuclear receptor, Peroxisome Proliferator Activated Receptor γ (*PPARγ*) in paired subcutaneous (SAT) and visceral (VAT) AT of obese subjects (n = 50) and to relate these values to serum concentrations of LEP and ADIPOQ (ii) evaluate the association of expression levels of marker genes in AT and serum with POP concentrations in AT.

**Results and Conclusions:**

Leptin and adiponectin levels in serum were positively correlated to respectively expression levels of leptin in SAT and adiponectin in VAT. Our study shows more significant correlations between gene expression of obesity marker genes and POP concentrations in VAT compared to SAT. Since VAT is more important than SAT in pathologies associated with obesity, this suggests that POPs are able to influence the association between obesity and the development of associated pathologies. Moreover, this finding reveals the importance of VAT when investigating the obesogen hypothesis. Concerning PPARγ expression in VAT, negative correlations with polychlorinated biphenyls (PCBs) concentrations were found in non T2D patients. LEP serum concentrations correlated with several PCBs in women whereas in men no correlations were found. This strengthens the potential importance of gender differences in obesity and within the obesogen hypothesis.

## Introduction

Persistent Organic Pollutants (POPs) are man-made chemicals that are environmentally persistent, leading to bioaccumulation and biomagnification in the food chain, an important exposure route for humans. POPs, including polychlorinated biphenyls (PCBs) and polybrominated diphenylethers (PBDEs), are accumulating in fatty compartments and in particular in adipose tissue (AT) due to their lipophilic character. Over the last decades, it has been shown that AT is not only a storage place for excessive energy, but that it acts as a metabolic and endocrine organ and it also has a protective role as storage place for POPs[1].

Since several POPs are described as endocrine disrupting compounds (EDCs), effects on AT, and more broadly on metabolic diseases such as obesity and diabetes are suspected[2]. The environmental obesogen hypothesis endorses this statement, stating that prenatal or lifetime exposure to environmental pollutants plays a role in the global obesity epidemic [3]. In this context, several *in vitro*, *in vivo* and epidemiological studies have investigated the potential link of POPs with obesity development [reviewed in [2] and[4]]. *In vitro* studies have indicated the induction of adipocyte differentiation and pre-adipocytes proliferation after PCB and/or DDE exposure[5], [6]. Moreover, *in vivo* rodent experiments have indicated a potential role of PBDEs and PCBs on obesity development[5], [7], [8]. Additionally, Lyche et al. (2010) [9] showed an increased weight gain in female zebrafish exposed to a mixture of POPs. Epidemiological studies investigating the link between POPs and obesity are in general focusing on relationships between POPs and waist-hip-ratio, BMI or other anthropometric measurements (reviewed in [4]).

However, information about the relationship between POP levels and molecular endpoints such as obesity marker gene expression is lacking. This mechanistic information will be crucial to further unravel the obesogen hypothesis. The nuclear receptor peroxisome proliferator activated receptor γ (PPARγ) is such a well described obesity marker gene, highly expressed in AT. PPARγ is activated by fatty acids, pharmacological ligands (e.g. thiazolidinediones) and other xenobiotics and is a major regulator of adipocyte differentiation. Several environmental obesogens are known to be PPARγ agonists, suggesting an important role of this receptor in the obesogen hypothesis[10], [11]. Adipokines, signaling molecules secreted by the adipose tissue, are also well known obesity markers involved in the pathogenesis of obesity-related diseases such as Type 2 diabetes (T2D) or cardiovascular diseases[12]. Leptin, for instance, controls food intake and energy expenditure and is increased in obese subjects[12], [13], whereas adiponectin has a protective role in the development of obesity-associated diseases and is decreased in serum of obese patients[12], [14]. The pro-inflammatory product, tumor necrosis factor α (TNFα) is a third important adipokine, since the inhibition of TNFα signaling in obese animals leads to an improvement of the insulin sensitivity[15], [16]. By implementing the measurement of these gene markers in epidemiological study designs, the link between marker expression and compound levels can be examined to further elucidate the role of chemicals, such as POPs, in obesity and obesity-related diseases.

This study is part of a larger trial to unravel the link between endocrine disruption by POPs and obesity. In total, 50 obese patients undergoing bariatric surgery were included, and paired samples of visceral adipose tissue (VAT) and subcutaneous adipose tissue (SAT) were examined for gene expression of obesity markers leptin, adiponectin and TNFα and PPARγ. In addition, adiponectin and leptin levels were measured in serum of those subjects. The aim of this study is therefore twofold: i) Evaluate depot specific differences in gene expression levels of obesity maker genes; ii) Assess, for the first time, associations between expression levels of obesity markers and POP concentration levels in SAT and VAT.

## Materials and Methods

### A. Study population

The Endorup trial is a prospective study with adult obese subjects conducted at the Weight Management Clinic of the Antwerp University Hospital. The aim of the trial is to unravel the hypothetical link between endocrine disruption by POPs and obesity. This trial was approved by the Ethical Committee of the Antwerp University Hospital (Belgian Registry number B30020097009) and registered at clinicaltrials.gov (number NCT01778868). All patients provided written informed consent and agreed to provide adipose tissue samples. Next to POP measurements in fat and serum, diabetic status and markers of body composition (BMI and amount of fat tissue) were gathered[17]. In participants without known history of T2D, an oral glucose tolerance test was performed and diabetic status was identified according to American Diabetes Association criteria. Weight and height were measured in the fasting state and undressed. A computed tomography (CT)-scan at the L4–L5 level was performed to assess the amount of VAT and SAT[18]. Data on POP concentration levels in VAT, SAT have recently been published by co-workers[17]. This study is a part of the aforementioned larger trial, but focusing on a subset of patients (n = 53), included between November 2009 and February 2012 and undergoing bariatric surgery at the Antwerp University Hospital. According to Belgian law, following criteria were required to be eligible candidates for bariatric surgery: 1) BMI≥40 kg/m^2^ or 2) BMI≥35 kg/m^2^ with at least one of the following co-morbidities: diabetes mellitus; obstructive sleep apnea or arterial hypertension, insufficiently controlled with 3 antihypertensive drugs. All patients underwent gastric bypass surgery, except for one female subject who underwent gastric banding. In total, paired VAT and SAT samples were gathered from 52 patients, of which 50 could be included with sufficient RNA quality for gene expression analysis.

### B. Fat and blood sampling

Fat samples were stored in glass vials at −20°C for POP analysis and immediately snap frozen and stored at −80°C for gene expression analysis. Serum samples were obtained in the fasting state prior to surgery and stored at −80°C for adipokine measurements.

### C. ELISA adipokine detection

Adiponectin and leptin protein concentrations were measured in serum using an enzyme-linked immunosorbent assay and manufacturer's instructions were followed (DuoSet ELISA, R&D Systems, Minneapolis, USA). Measurements were performed in duplicate and a standard was included in each 96 well plate. A 4-parameter logistic curve fit of the adipokine standards was performed to recalculate the adipokine concentration values using the Graphpad Prism 6.00 software. To measure samples within the range of the standard (leptin: 125–8000 pg/mL; adiponectin: 62.5–4000 pg/mL) serum samples were diluted 1/100 and 1/10000 for leptin and adiponectin quantification respectively. Recoveries of the internal standard for leptin and adiponectin were respectively 105% and 110%.

### D. RNA extraction and gene expression analysis

RNA was extracted from 100 mg AT using the RNeasy Lipid Tissue Mini Kit (Qiagen, Antwerp, Belgium) following the manufacturer's instructions. RNA purity and quality were evaluated using the NanoDrop spectrophotometer (NanoDrop Technologies, Montchanin, DE, USA), integrity was checked using agarose gel electrophoresis. A starting amount of 1 µg RNA was transcribed to first strand cDNA according to Revert Aid™ H Minus First strand cDNA synthesis kit for RT-PCR (Thermo Fisher Scientific, Zellik, Belgium). Real-time PCR reaction master mix was used following the manufacturer's instructions, starting with 50 ng of cDNA (Brilliant® II SYBR® Green QPCR master mix, Agilent Technologies, Santa Clara, CA). Template standards and primers were obtained from OriGene Technologies (Rockville, MD) for every measured gene: *PPARγ* (HK210365), *TNFα* (HK208349), *ADIPOQ* (HK209573) and *LEP*(HK204316). TATA box binding protein (*TBP*; HK209260) was used as a housekeeping gene. Gene expression values of the genes of interest (GOI) were subsequently normalised to the housekeeping gene and therefore expressed as gene expression value of GOI/TBP, amplified by a factor of 1000.

### E. Analyses of POPs

POP measurements in VAT and SAT were performed by the Toxicological Centre (University of Antwerp). In all samples 28 PCB congeners and 5 PBDEs were targeted for analysis. A full list with IUPAC no and concentration levels of the compounds measured in AT is shown in Table S1. The analytical methods, quality assurance and quality control have been published previously [17].

### F. Statistical analysis

Statistical analysis was performed using SPSS, version 20.0 (SPSS, Chicago, IL). Levels of contaminants below the limit of quantification (LOQ) were entered in the database as ½×LOQ. Normality of distribution was verified using the Shapiro-Wilk test. POPs and gene expression levels displayed a skewed distribution, which was not transformable to normality. Therefore, a Mann-Whitney U test was performed to test for significant differences between 2 groups and results were considered significant at p≤0.05. Additionally, Spearman rank correlation was used to test for associations between parameters. Correlations were considered significant at p≤0.05, additionally a Bonferroni correction for multiple testing was applied for these analyses resulting in a stricter significance level of 0.007. The compounds that accounted for more than 50% of the PCB (CB138, CB153 and CB180) and PBDE (BDE47, BDE153) burden were selected for further statistical analysis.

## Results

### A. Expression levels of obesity markers in relation to anthropometric parameters

General characteristics of the 50 obese patients under study, such as age, gender, BMI, diabetic status, amount of VAT and SAT and VAT/SAT ratio are shown in Table 1.

**Table 1:** Characteristics of the study population.

Ethnicity	
**Caucasian subjects**, n	49
**non-Caucasian subjects**, n	1
**Gender**	
**Men**, n	17
**Women**, n	33
**Diabetic status**	
**Subjects with T2D**, n	8
**Subjects without T2D**, n	42
**Age (years)**, median (min-max)	40.50 (18–58)
**BMI (kg/m^2^)**, median (min-max)	41.15 (35.60–51.40)
**VAT/SAT ratio**, median (min-max)	0.27 (0.09–0.90)
**CT visceral AT (cm^2^)**, median (min-max)	192 (51–481)
**CT subcutaneous AT (cm^2^)**, median (min-max)	675 (329–1055)
**CT total AT (cm^2^)**, median (min-max)	904 (540–1334)

Ethnicity, gender and diabetic status of the subjects are represented as well as clinical and anthropometric characteristics which are represented as median values (minimum–maximum). All included subjects were citizens of Belgium. *T2D = Type 2 diabetes; BMI = body mass index; VAT/SAT = ratio visceral/subcutaneous adipose tissue; CT = Computed tomography*.

In this study, gene expression values of the obesity marker genes leptin, adiponectin, TNFα and PPARγ were measured in paired SAT and VAT samples of 50 obese patients. In addition, leptin and adiponectin hormone expression levels were measured in serum samples of these patients. An overview of the median expression levels (with minimum and maximum levels) in both fat depots and the hormone levels in serum are presented in Table 2. For all gene markers, expression levels were higher in SAT than in VAT, although this was not significant for TNFα (Table 2).

**Table 2:** Expression levels of obesity marker genes.

	Gene expression				Serum concentration
	SAT Median (min-max)	VAT Median (min-max)	p-value		µg/mL Median (min-max)
**Leptin (** ***LEP*** **)**	549 (3.08–4100)	209 (3.24–1030)	**0.004**	**Leptin (LEP)**	0.101 (0.03–0.21)
**Adiponectin (** ***ADIPOQ*** **)**	4660 (813–34900)	2920 (510–11200)	**0.014**	**Adiponectin (ADIPOQ)**	10.9 (1.86–53.1)
**TNFα**	3.22 (0.01–17.4)	2.53 (0.16–16.3)	0.679		
**PPARγ**	852 (202–14500)	553 (242–1890)	**0.000**		

Data represent the median gene expression levels of the marker genes normalized to the household gene TATA box binding Protein (*TBP*) expression in SAT and VAT and the median values of the serum concentration levels of adiponectin and leptin. Minimum and maximum expression ratios are represented between brackets. Differences between VAT an SAT gene expression levels were examined with a Mann Whitney U-test, significant differences (p≤0.05; n = 50) are shown in bold. *SAT = Subcutaneous adipose tissue; VAT = Visceral adipose tissue*.

**Figure 1:**
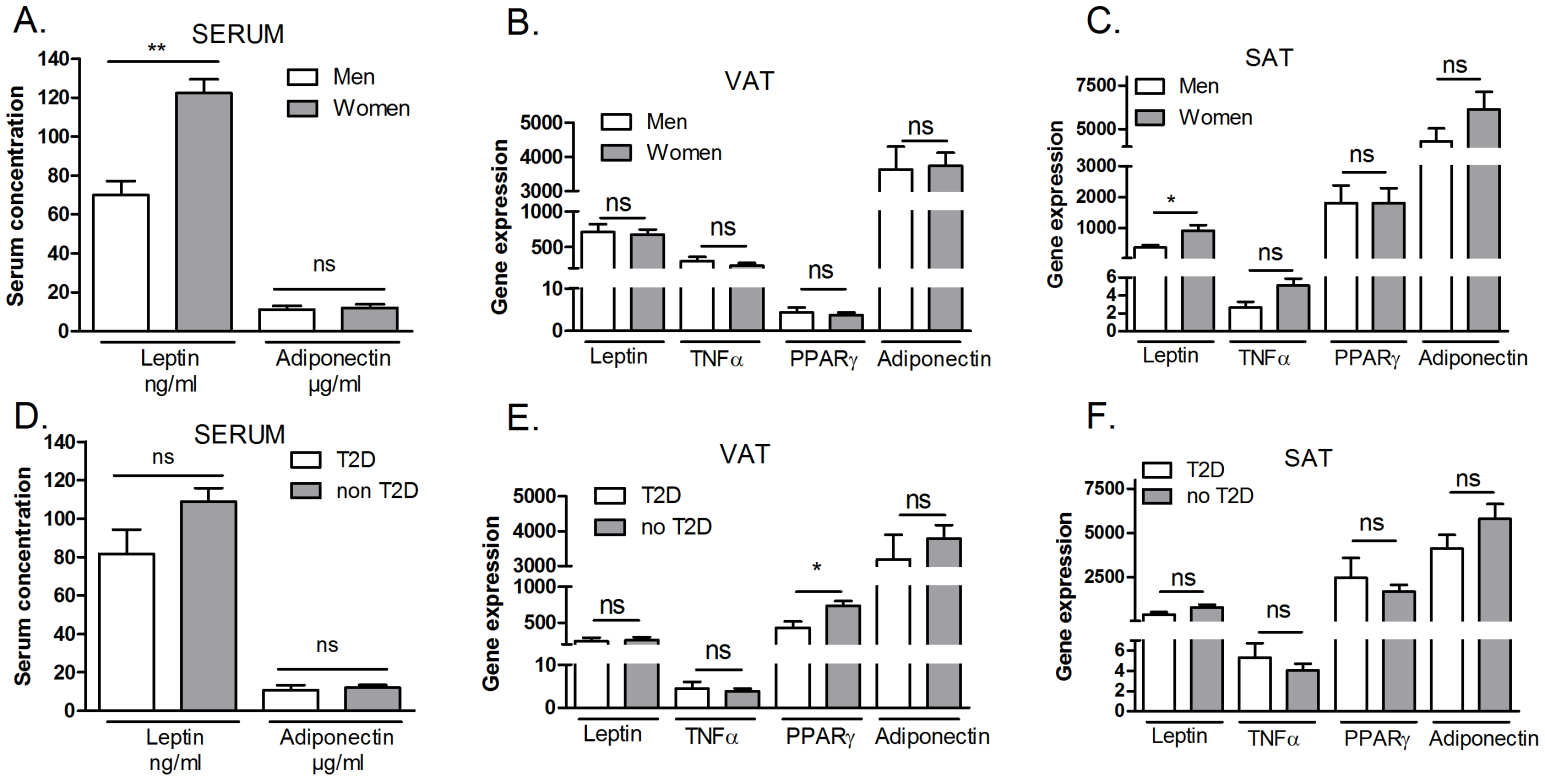
Serum concentrations and gene expression levels of obesity markers in relation to gender and diabetes. Data represent mean (±SE) of the serum concentrations of leptin and adiponectin (A) and gene expression of leptin, adiponectin, TNFα and PPARγ normalised to the gene expression of TBP gene*1000 of AT samples (B, C, D, E). Significant differences between men (n = 17) and women (n = 33) (B, C), diabetic (n = 8) and non diabetic patients (n = 42) (D, E) are indicated with asterisks (Mann Whitney U-test; *p≤0.05; **p≤0.01).

In a next step, expression levels of these obesity markers in VAT, SAT and serum were examined according to gender and diabetic status, two important influencing parameters in obesity (Figure 1).

In serum, significantly higher levels of leptin were measured in women, compared to men (p<0.001; Figure 1A). Moreover, in SAT the same gender differences were observed for gene expression levels of leptin (p = 0.029; Figure 1C). In this cohort of 50 patients, 8 diabetic obese patients were included and therefore diabetic status could be assessed as influencing factor. Only PPARγ expression in VAT differed between diabetic (T2D) and non diabetic patients (non T2D), with a lower expression in T2D patients (p = 0.016; Figure 1E). Other gene expression levels did not differ depending on gender or diabetic status (Figure 1). Since these data show that gender and diabetic status are important influencing factors for certain obesity markers, the influence of these factors was particularly examined in all further analyses as surplus to the overall effects on the whole patient population.

Finally, estimates of body composition, such as BMI, the total fat area (CT_total_) and VAT/SAT ratio (Table 3 and 4), were examined for their relation to the obesity marker expression levels. In the entire cohort, TNFα and leptin expression in VAT was positively correlated with the VAT/SAT ratio, whereas leptin in serum was negatively correlated with this parameter (Table 3). Looking in more detail to the influence of gender for leptin expression (in serum and SAT) shows that the overall negative correlation of leptin in serum with VAT/SAT ratio in the entire cohort is due to the strong negative correlation of leptin in serum with VAT/SAT in women (ρ = −0.65, p<0.001; Table 4), whereas this relation could not be established in men. Adiponectin gene expression in both fat depots was negatively correlated with the CT_total_, whereas PPARγ was positively correlated with the CT_total_ (Table 3). The positive relationship between PPARγ and the CT_total_ disappeared when separate groups of diabetic and non diabetic patients were considered (Table 4). BMI did not correlate with the expression of any obesity marker (Table 3 and 4).

**Table 3:** Spearman correlation coefficients (ρ) of total CT, VAT/SAT ratio and BMI with obesity marker expression levels in fat tissue (gene expression values) and serum (adipokine concentrations) in the overall population (n = 50).

		CT total		VAT/SAT		BMI	
		ρ	p-value	ρ	p-value	ρ	p-value
**SERUM**	**Leptin (LEP)**	0.072	0.622	**−0.388**	**0.006**	0.204	0.155
	**Adiponectin (ADIPOQ)**	−0.177	0.223	−0.250	0.084	−0.147	0.307
**VAT**	**Leptin (** ***LEP*** **)**	−0.153	0.293	**0.368**	**0.009**	−0.106	0.466
	***TNFα***	−0.080	0.586	**0.333**	**0.019**	0.033	0.819
	***PPARγ***	**0.289**	**0.044**	−0.127	0.385	0.204	0.155
	**Adiponectin (** ***ADIPOQ*** **)**	**−0.314**	**0.028**	−0.168	0.249	−0.145	0.317
**SAT**	**Leptin(** ***LEP*** **)**	−0.277	0.054	−0.055	0.705	−0.005	0.972
	***TNFα***	−0.185	0.203	−0.002	0.989	−0.156	0.280
	***PPARγ***	−0.059	0.687	0.006	0.967	−0.011	0.938
	**Adiponectin (** ***ADIPOQ*** **)**	**−0.314**	**0.028**	−0.075	0.610	−0.026	0.859

Data represent ρ values and significance level (p-value). Significant values (p-value≤0.05) are indicated in bold. *T2D = Type 2 diabetes; VAT/SAT = ratio visceral/subcutaneous adipose tissue; SAT = Subcutaneous adipose tissue; VAT = Visceral adipose tissue.*

**Table 4:** Spearman correlation coefficients (ρ) of total CT, VAT/SAT ratio and BMI with obesity marker expression levels in fat tissue (gene expression values) and serum (adipokine concentrations) related to gender and diabetic status.

			n	CT total		VAT/SAT		BMI	
				ρ	p-value	ρ	p-value	ρ	p-value
**SERUM**	**Leptin (LEP)**	M	17	0.418	0.107	0.044	0.871	0.439	0.078
		F	33	0.068	0.706	**−0.650**	**<0.001**	0.113	0.531
**SAT**	**Leptin (** ***LEP*** **)**	M	17	−0.306	0.249	−0.124	0.649	−0.017	0.948
		F	33	−0.282	0.112	−0.013	0.941	−0.058	0.751
**VAT**	***PPARγ***	T2D	8	0.643	0.086	0.524	0.183	0.429	0.289
		non T2D	42	0.265	0.095	−0.006	0.971	0.139	0.379

Data represent ρ values and significance level (p-value). Significant values (p-value≤0.05) are indicated in bold. *T2D = Type 2 diabetes; VAT/SAT = ratio visceral/subcutaneous adipose tissue; SAT = Subcutaneous adipose tissue; VAT = Visceral adipose tissue.*

### B. Expression levels of the marker genes in visceral and subcutaneous fat depots

For adiponectin and leptin, relationships between serum concentrations and AT gene expression levels were analyzed. Adiponectin serum concentrations were correlated with gene expression values in VAT (ρ = 0.347; p-value = 0.013), whereas leptin serum levels correlated with gene expression in SAT (ρ = 0.293; p-value = 0.039). The association between leptin in SAT and serum disappeared when male and female patients were analyzed separately (data not shown).

Additionally for all genes, relationships between VAT and SAT gene expression were analyzed. However, only adiponectin gene expression was correlated between both AT depots (ρ = 0.415; p-value<0.001).

### C. Relation between obesity marker levels and the POP concentration levels in fat

**Figure 2:**
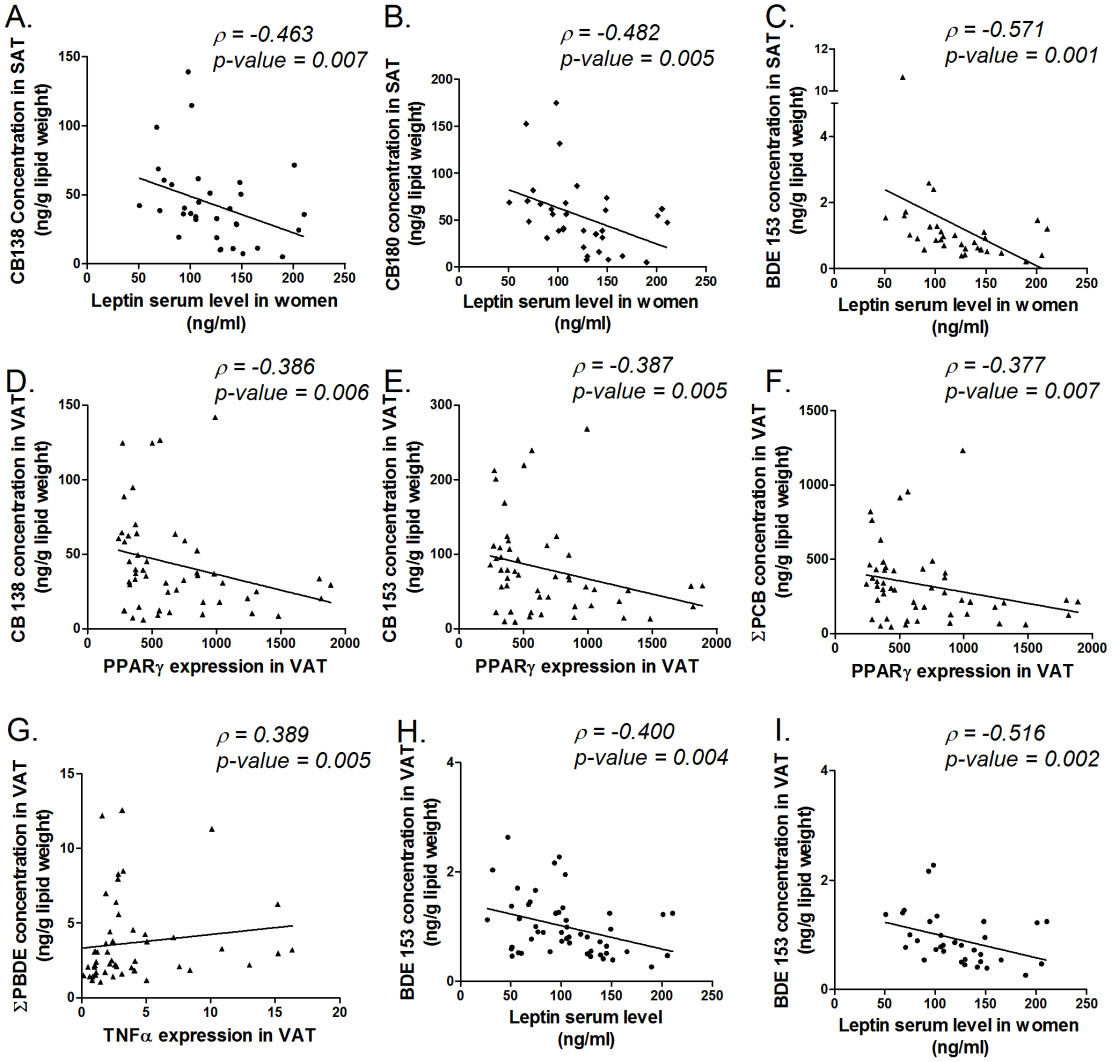
Correlations between obesity marker genes and POP concentrations in AT. Scatterplots are representing gene expression values of obesity maker genes (X-axis) in relation to the POP concentrations (Y-axis). Only highly significant spearman correlations (p≤0.007) are shown, ρ-values and p-values of the Spearman correlations are indicated on the scatterplot.

In this paper, associations between expression of obesity markers (adiponectin, leptin, TNFα and PPARγ) and PCBs and PBDEs in adipose tissue are discussed (Table 5), highly significant correlations (p≤0.007) are shown in scatter plots on Figure 2. In this paper, only the most abundant compounds, accounting for more than 50% of the PCB (CB138, CB153 and CB180) and PBDE (BDE47, BDE153) burden were discussed in detail. In the supplemental material, associations with all individual PCB and PBDE congeners are given (Table S2). Additionally, associations between obesity markers and POP serum levels were evaluated. Similar associations were found compared to AT POP concentrations, therefore these correlations are not discussed in detail in this paper (Table S3).

**Table 5:** Spearman correlations coefficients of POP levels in AT with serum concentrations (leptin and adiponectin) and gene expression in fat tissue.

			VAT						
		***n***	**CB138**	**CB153**	**CB180**	**ΣPCB**	**BDE47**	**BDE153**	**ΣPBDE**
	**Leptin (LEP)**	*50*	−0.148	−0.156	−0.230	−0.194	−0.099	**−0.400**	**−0.329**
			(0.304)	(0.281)	(0.108)	(0.176)	(0.492)	**(0.004)**	**(0.020)**
**SERUM**	Leptin (LEP) M	*17*	−0.027	−0.047	−0.064	−0.049	−0.225	−0.179	−0.277
			(0.918)	(0.859)	(0.808)	(0.852)	(0.384)	(0.492)	(0.282)
	Leptin (LEP) F	*33*	**−0.424**	**−0.381**	**−0.437**	**−0.395**	−0.040	**−0.516**	**−0.371**
			**(0.014)**	**(0.029)**	**(0.011)**	**(0.023)**	(0.826)	**(0.002)**	**(0.034)**
	**Adiponectin (ADIPOQ)**	*50*	−0.240	−0.163	−0.149	−0.196	−0.050	−0.124	−0.135
			(0.093)	(0.258)	(0.300)	(0.172)	(0.732)	(0.389)	(0.351)
	**Leptin (** ***LEP*** **)**	*50*	0.235	0.260	**0.315**	0.249	0.220	**0.335**	**0.286**
			(0.101)	(0.068)	**(0.026)**	(0.081)	(0.124)	**(0.017)**	**(0.044)**
	***TNFα***	*50*	0.043	0.051	0.099	0.062	**0.359**	**0.289**	**0.389**
			(0.767)	(0.723)	(0.495)	(0.667)	**(0.010)**	**(0.042)**	**(0.005)**
**VAT**	***PPARγ***	*50*	**−0.386**	**−0.387**	**−0.356**	**−0.377**	0.040	−0.277	−0.159
			**(0.006)**	**(0.005)**	**(0.011)**	**(0.007)**	(0.783)	(0.051)	(0.271)
	*PPARγ* T2D	*8*	0.452	0.381	**0.714**	0.381	−0.048	0.357	0.071
			(0.260)	(0.352)	**(0.047)**	(0.352)	(0.911)	(0.385)	(0.867)
	*PPARγ* NON T2D	*42*	**−0.308**	**−0.331**	**−0.306**	**−0.322**	0.067	−0.201	−0.096
			**(0.047)**	**(0.032)**	**(0.048)**	**(0.038)**	(0.675)	(0.202)	(0.547)
	**Adiponectin (** ***ADIPOQ*** **)**	*50*	**−0.278**	−0.248	−0.219	0.268	−0.026	−0.192	−0.166
			**(0.050)**	(0.083)	(0.127)	(0.059)	(0.858)	(0.182)	(0.249)

			SAT						
		***n***	**CB138**	**CB153**	**CB180**	**ΣPCB**	**BDE47**	**BDE153**	**ΣPBDE**
	**Leptin (LEP)**	*50*	−0.160	−0.162	−0.240	−0.211	−0.031	**−0.374**	−0.174
			(0.266)	(0.261)	(0.093)	(0.141)	(0.830)	**(0.008)**	(0.226)
**SERUM**	Leptin (LEP) M	*17*	−0.039	−0.037	−0.088	−0.044	−0.135	−0.031	0.069
			(0.881)	(0.889)	(0.736)	(0.866)	(0.606)	(0.907)	(0.794)
	Leptin (LEP) F	*33*	**−0.463**	**−0.423**	**−0.482**	**−0.445**	−0.098	**−0.571**	**−0.359**
			**(0.007)**	**(0.014)**	**(0.005)**	**(0.009)**	(0.586)	**(0.001)**	**(0.040)**
	**Adiponectin (ADIPOQ)**	*50*	−0.234	−0.152	−0.128	−0.182	0.001	−0.055	−0.016
			(0.102)	(0.293)	(0.374)	(0.207)	(0.993)	(0.704)	(0.914)
	**Leptin (** ***LEP*** **)**	*50*	−0.024	−0.064	−0.096	−0.050	0.262	−0.081	0.043
			(0.866)	(0.660)	(0.506)	(0.730)	(0.066)	(0.576)	(0.767)
	Leptin (*LEP*) M	*17*	−0.306	−0.328	−0.287	−0.292	−0.252	−0.351	−0.265
			(0.232)	(0.198)	(0.264)	(0.256)	(0.328)	(0.168)	(0.305)
**SAT**	Leptin (*LEP*) F	*33*	0.029	0.005	−0.030	0.035	**0.379**	0.074	0.153
			(0.874)	(0.976)	(0.867)	(0.846)	**(0.030)**	(0.682)	(0.395)
	***TNFα***	*50*	0.054	0.020	−0.058	−0.005	0.171	−0.025	0.085
			(0.709)	(0.891)	(0.690)	(0.974)	(0.235)	(0.863)	(0.556)
	***PPARγ***	*50*	0.002	−0.048	−0.067	−0.060	−0.243	−0.142	−0.220
			(0.989)	(0.742)	(0.642)	(0.678)	(0.090)	(0.326)	(0.125)
	**Adiponectin (** ***ADIPOQ*** **)**	*50*	−0.083	−0.090	−0.103	−0.074	−0.087	−0.212	−0.181
			(0.569)	(0.536)	(0.477)	(0.610)	(0.549)	(0.139)	(0.208)

Data represent ρ values (significant values are shown bold: p≤0.05; bold and underlined: p≤0.007) M: Men; F: Women.

Concerning POP concentrations in VAT, leptin gene expression was positively correlated with concentrations of BDE153, ΣPBDE and CB180 (Table 5). TNFα expression levels in VAT were positively correlated with BDE47, BDE153 and ΣPBDE (Table 5). Concerning PPARγ expression in VAT, negative correlations were found with all PCB congeners in VAT (Table 5). Analysis of the cohort separately, taking the diabetic status into account, revealed a negative correlation between PPARγ and CB138, CB153 and ΣPCB in patients with normal glucose tolerance, whereas a positive correlation was noticed between PPARγ gene expression and CB180 concentration in VAT of T2D-patients (Table 5). Adiponectin gene expression was negatively correlated with CB138 (Table 5).

Additionally, associations between POP concentrations in VAT with serum levels of leptin and adiponectin were assessed (Table 5). In the entire cohort, a negative association between leptin serum levels and BDE153 and ΣPBDE levels was detected. However, when analyzing the two sexes separately, all chlorinated compounds and some brominated compounds (BDE153, ΣBDE) in women were negatively correlated with leptin serum concentration, while in men, no POP was correlated with leptin serum concentration (Table 5). Considering adiponectin serum concentration, no associations with POP concentrations in VAT were found (Table 5).

In SAT, considering the whole population, none of the gene expression levels of all measured genes were correlated with the measured POPs levels (Table 5). For leptin gene expression, a separate analysis was performed depending on gender. In men, no correlations were observed with POP levels in SAT, whereas in women, only BDE47 positively correlated with leptin gene expression levels (Table 5).

Similar to VAT, associations between POP concentrations in SAT and leptin or adiponectin concentrations in serum were also analyzed (Table 5). Concerning adiponectin serum concentration, no correlations with POP concentrations in SAT were found (Table 5). For leptin, analysis of the whole cohort, reveals a negative association with BDE153. The analysis of men and women separately revealed negative correlations between serum concentrations of leptin with all analyzed PCBs, BDE153, and ΣPBDE in SAT from women, whereas in men, no correlations were found (Table 5).

Linear regression analysis was performed with all obesity marker genes as dependent variables and the POPs in adipose tissues as independent variables adjusted for BMI and diabetes or gender (Table 6). Only models with leptin serum level as dependent variable and adjusted for gender and BMI were significant. Moreover, in significant models, BMI never reached significance, whereas the levels of CB180, ΣPCB, BDE153 in SAT and CB180 and ΣPCB in VAT did. This emphasizes the gender dependency of the POP influence on leptin serum concentrations.

**Table 6:** Overview of standard linear regression analyses with obesity marker levels as dependent variables and POP levels in VAT/SAT, BMI and gender or diabetes as independent variables.

DEPENDENT VARIABLE	INDEPENDENT VARIABLE			MODEL	
**SAT**		**β**	**p-value**	**R^2^ adjusted**	**p-value**
	CB138	−0.223	0.063	0.330	<0.001
	**Gender**	**−0.571**	**<0.001**		
	BMI	0.106	0.368		
	CB153	−0.227	0.058	0.332	<0.001
	**Gender**	**−0.563**	**<0.001**		
	BMI	0.105	0.373		
	**CB180**	**−0.268**	**0.024**	0.354	<0.001
	**Gender**	**−0.561**	**<0.001**		
	BMI	0.104	0.369		
	**ΣPCB**	**−0.257**	**0.032**		
**Leptin (LEP) serum**	**Gender**	**−0.567**	**<0.001**	0.347	<0.001
	BMI	0.118	0.315		
	BDE47	0.023	0.855		
	**Gender**	**−0.553**	**<0.001**	0.278	<0.001
	BMI	0.098	0.427		
	**BDE153**	**−0.256**	**0.035**		
	**Gender**	**−0.564**	**<0.001**	0.345	<0.001
	BMI	0.053	0.655		
	ΣPBDE	−0.101	0.407		
	**Gender**	**−0.560**	**<0.001**	0.288	<0.001
	BMI	0.097	0.427		
**VAT**		**β**	**p-value**	**R^2^ adjusted**	**p-value**
	CB138	−0.229	0.057	0.332	<0.001
	**Gender**	**−0.569**	**<0.001**		
	BMI	0.104	0.377		
	CB153	−0.225	0.061	0.331	<0.001
	**Gender**	**−0.561**	**<0.001**		
	BMI	0.101	0.390		
	**CB180**	**−0.248**	**0.038**		
	**Gender**	**−0.556**	**<0.001**	0.343	<0.001
**Leptin (LEP) serum**	BMI	0.101	0.390		
	**ΣPCB**	**−0.250**	**0.037**	0.343	<0.001
	**Gender**	**−0.561**	**<0.001**		
	BMI	0.114	0.332		
	BDE 47	0.025	0.836		
	**Gender**	**−0.554**	**<0.001**	0.278	<0.001
	BMI	0.098	0.431		
	BDE153	−0.238	0.056		
	**Gender**	**−0.505**	**<0.001**	0.333	<0.001
	BMI	0.059	0.619		

Only significant models are shown, and the adjusted R^2^ and p-value (n = 50) of each model are shown in the table. Standardized coefficients are reported (β), with their p-value. Significant variables in a model are shown in bold. No significant model was obtained by including diabetic status as independent variable.

## Discussion

**Figure 3:**
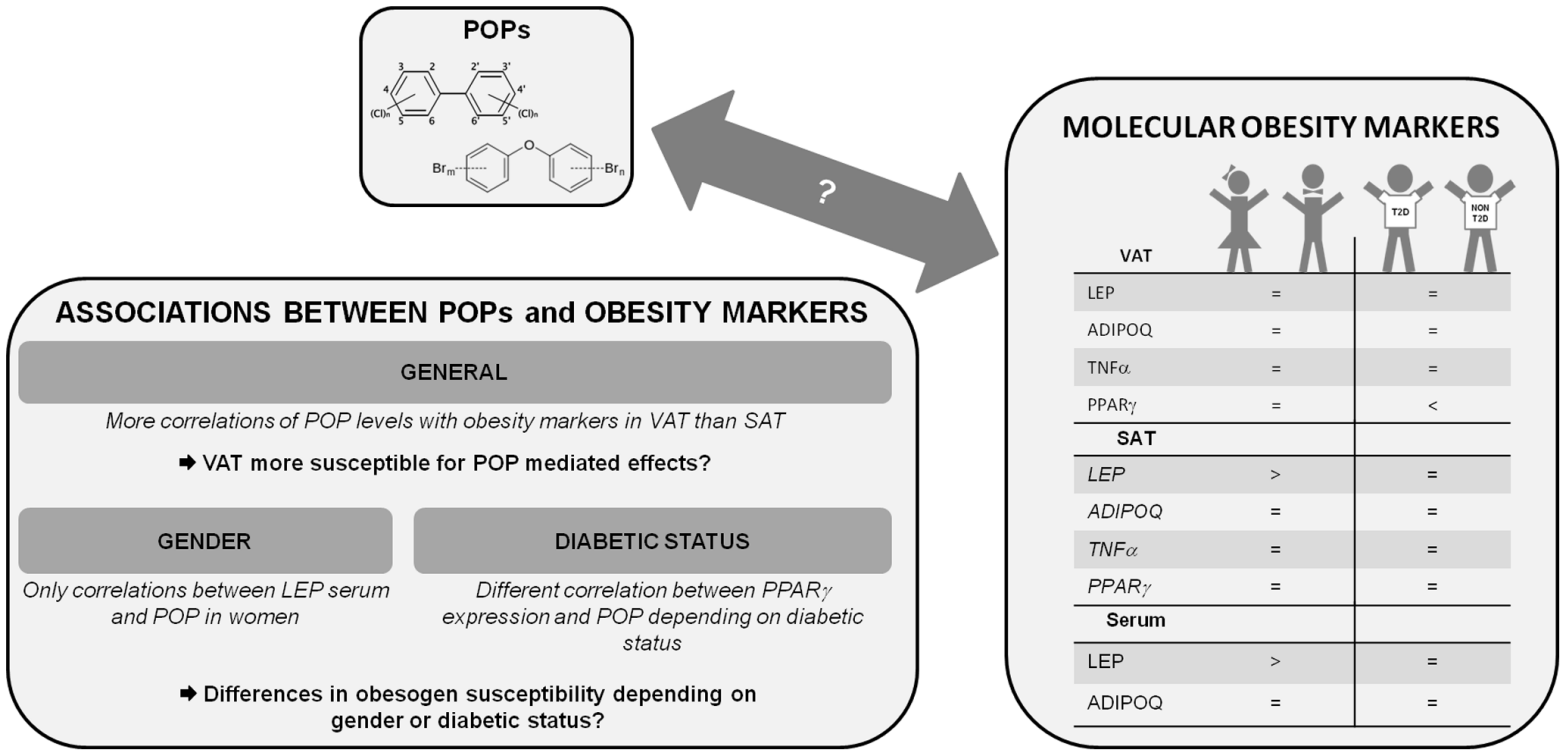
Overview of the major findings of this manuscript. A. Levels of obesity markers in VAT, SAT and serum of the obese population depending on gender and diabetic status. B. Associations between obesity markers and POP levels.

The environmental obesogen hypothesis states that exposure to environmental pollutants early in life or throughout life time has an influence on obesity development [3], [19]. A dose-response relationship between POP concentrations, and metabolic syndrome or diabetes has previously been shown by Lee and colleagues [20], [21]. However, to our knowledge, this is the first study to investigate the associations between the gene expression of important obesity markers (adipokines and PPARγ) in AT and POP concentrations in fat from obese patients. To give an overview of the major findings of this study, a summarizing figure was made consisting of the differences in expression levels of the obesity marker genes depending on gender and diabetic status (Figure 3A) and the conclusions concerning the POP-obesity marker associations (Figure 3B).

### A. Gene expression in SAT and VAT of obese patients

VAT in comparison to SAT fat deposition is known to correlate to metabolic complications, such as cardiovascular diseases, hypertension and T2D [22]. Since only a few studies have been conducted with paired VAT and SAT tissues, the depot dependent differences in gene expression of maker genes was evaluated. The gene-expression levels of adiponectin and leptin were higher in SAT compared to VAT, confirming the previous studies with obese patients [23]–[26]. However, this was not true for TNFα levels which were similar in both fat depots, also as previously seen [27]. PPARγ expression was higher in SAT compared to VAT depot. This finding is in contradiction with another study that did not find differences between fat depots of obese people [28], but in accordance with Lefebvre et al. (1998) [25] that reported the same differential expression, especially in lean subjects. Moreover, the previously mentioned studies included a rather small set of patients (n = 6–10) compared to our study (n = 50).

Our recent understanding of the functions of adipose tissue have emphasized not only its role in energy storage, but also in the regulation of complex metabolic and endocrine functions. In this context, leptin and adiponectin, the two most abundant adipokines produced by white AT, represent one of the best examples of adipocytokines involved in the control of energy expenditure, lipid and carbohydrate metabolism. Leptin and adiponectin secretion is counter-regulated *in vivo* since plasma leptin concentrations are significantly elevated in obese subjects in proportion to BMI, while adiponectin secretion decreases in relation to the amount of AT. Therefore, the association of the VAT/SAT deposition with the serum concentration of these valuable markers of adiposity was also examined in this study. A positive correlation was found between leptin serum levels and its gene expression in SAT, whereas VAT/SAT ratio negatively correlated with the leptin concentration in serum of women (Table 3). These findings are in concordance with Garaulet et al. (2000) [29] who have shown that in women higher leptin concentrations in serum were associated with larger subcutaneous, but not visceral CT values, indicating the importance of SAT for leptin production. This finding could indicate that women who are viscerally obese have lower levels of leptin, potentially causing a broken feedback loop on energy intake [30]. Concerning adiponectin, only VAT expression correlated with its serum concentration, indicating the possible important role of VAT for adiponectin production. Previous studies have shown both concordant and discordant results: some studies found a positive correlation between SAT gene expression and serum concentration of adiponectin [31], whereas others did not find a significant association in SAT, in concordance with our results[32]. Perrini et al.[33] show that rates of adiponectin secretion were threefold higher in human adipocytes from VAT differentiated *in vitro* compared to adipocytes from SAT, confirming our results. The discrepancy between our results showing a higher adiponectin gene expression in SAT compared to VAT, but a correlation between adiponectin serum concentration and VAT content, indicate that adiponectin gene and protein expression may be regulated in a different manner in both fat depots.

Gender differences are important in the context of insulin resistance, body composition and energy balance [34]. Men accumulate more visceral fat, leading to a more apple-shaped body fat distribution that is linked with deleterious metabolic consequences. In contrast, women have in general more body fat than men, but tend to accumulate in a pear-shaped body fat distribution. The mechanisms behind the regulation of fat distribution are possibly intrinsic gender differences between pre-adipocytes and adipocytes or due to the modulatory role of sex steroids or environmental factors[35]. Therefore, differences in gene expression and adipokine serum concentration levels between both sexes were tested. In concordance with previous findings, our results indicate a higher leptin expression (in SAT) and higher leptin serum concentrations in women compared to men[34]. Previous studies reported higher levels of adiponectin in women compared to men and a more marked difference in lean compared to obese subjects [36]. In our study, no significant differences were found for adiponectin levels, probably due to the lack of lean subjects in our cohort.

PPARγ agonists, such as thiazolidinediones, are used as therapeutic agents in T2D treatments, reversing the insulin resistance in target tissues [37]. Interestingly, in accordance with the literature, diabetic patients had lower PPARγ expression in VAT compared to patients with normal glucose tolerance[38], [39].

### B. Relation between obesity marker genes in adipose tissue and POPs

Considering the gene expression of marker genes in general, more correlations with POPs were found in VAT compared to SAT (Table 5). This might suggest that VAT is more sensitive for POP dependent effects.

In VAT, leptin gene expression was positively correlated with key POPs, such as CB180 and BDE153, indicating a potential triggering effect on leptin expression in VAT depot. TNFα expression in VAT was positively correlated with levels of PBDEs, suggesting a comparable triggering effect on the gene expression of this inflammatory cytokine. Adiponectin expression in VAT correlated negatively with CB138, indicating that POPs could have an influence on obesity-related disorders by suppressing the expression of adiponectin, the protective adipokine. This is in concordance with the *in vitro* effects of another PCB congener, CB77, suppressing the adiponectin expression of mature adipocytes[5]. Considering PPARγ expression in VAT, distinctive correlations were found depending on the diabetic status of the patients. In non diabetic patients, the concentrations of several PCBs were negatively correlated with PPARγ expression, whereas in diabetic patients, a positive association was found between PPARγ gene expression and CB180 concentration. This finding could indicate possible POP dependent differences in diabetic patients. However, it should be noted that our subset of diabetic patients is rather small (n = 8).

There is only one previous paper reporting the association between elevated POP levels in AT and the increased expression of some POP-targeted genes, such as targets of the Aryl hydrocarbon receptor (AhR) and genes involved in low-grade inflammation[40]. Nevertheless, the associations between the expression of the two major adipokines in different fat compartments and the POP levels in AT is novel.

### C. Relation between obesity markers in serum and POPs in AT

Leptin and adiponectin are released into serum after local production in adipose tissue: the association between adipokine serum concentrations and POP levels in an obese human population has only been evaluated in one study revealing a negative correlation between CB153 and adiponectin levels in blood of women[41]. In contrast, our results indicate no relation between adiponectin serum levels and POP concentrations in AT.

Considering leptin serum levels, clear gender dependent effects of POPs were observed. Our study revealed a negative association between leptin serum concentrations in women, and the levels of several PCBs and BDE153 in both fat depots. Remarkably, in men, none of the analysed POPs correlated with leptin serum concentration (Table 5). This was further confirmed by the linear regression analysis, indicating the importance of gender and certain POP levels for leptin serum concentration prediction. Interestingly, BMI was not a predicting variable for this obesity marker in our cohort. However, it should be noted that this study consists of subjects with a rather narrow BMI range (Table 1). This suggests that in women leptin levels could be more influenced by POPs compared to men, indicating a new aspect in the obesogen hypothesis and stressing the importance of gender differences in terms of the POP-adipokine relationships. In accordance with our findings, other authors have indicated that prenatal POP exposure might have a greater impact on the weight development of girls compared to boys[4]. Interestingly a study of co-authors on the same cohort, describing the distribution of POPs in both fat depots, did not reveal differences in POP accumulation between AT of men and women[17].

These negative associations (in women) seem to be in disagreement with the positive association between POP concentrations and VAT levels of leptin. However, this discrepancy could be explained by the importance of SAT for leptin production. Therefore, the effects of POPs in VAT may only have a moderate effect on the final leptin serum concentrations.

Even though this study is of major importance since it is the first one studying the potential associations between POP concentrations and levels of obesity markers, it should be noted that overall the sample size is limited and that these findings need to be confirmed in a broader study group. This study is therefore a preliminary study indicating the importance of measuring obesity markers together with POP concentrations, to further unravel the obesogen hypothesis.

### D. POPs as obesogens?

Although the approach of this study is novel and offer a more detailed view of POPs and obesity in a human population, more mechanistic studies are needed to further elucidate potential obesogenic mechanisms of action of these compounds. Animal studies have indicated the importance of exposure during developmental periods, implicating possible epigenetic changes due to obesogen exposure. Indeed developmental exposure to the PCB mixture Aroclor 1254 was associated with an increased body weight of the mouse pups on post natal days 16–20 [42]. But also adult exposure may be important, as suggested by the study of Arscenescu et al. (2008) where exposure to PCB-77 in adult mice leaded to an AhR-dependent increase in body mass [5]. Concerning PBDEs, Lilienthal et al. (2006) showed that pre-natal exposure to BDE-99 increased mouse birth weight [43]. Moreover, Suvorov et al. (2009) indicated that pre- and postnatal exposure to BDE-47 increased rat body weights from birth to puberty [44]. No mechanisms of obesogenicity have previously described for PBDEs, indicating the need for more mechanistic studies investigating the obesogenic mechanisms of action of POPs.

## Conclusions

The correlations between POPs in AT and leptin serum levels were only found in women. This emphasizes the impact of gender on the susceptibility to the obesogenic action of POPs. In general, more correlations between POPs and gene expression levels were found in VAT. We speculate that VAT could be more sensitive for POPs compared to SAT. Considering the previously reported importance of VAT within the development of obesity-related diseases, we suggest that POPs might influence the gene expression for adipokines in VAT and in that way, have an impact on the development of these metabolic disorders.

## Supporting Information

Table S1 **POP concentration levels in SAT/VAT.** Data represent the median concentration in ng/g lipid weight and the number of samples below the detection limit. Minimum, maximum concentrations of each compound are shown between brackets. The grey cases indicate compounds that account for at least 50% of the PCB or PBDE burden; these were further analyzed in this paper. *SAT = Subcutaneous adipose tissue; VAT = Visceral adipose tissue*.
(PDF)

Table S2 **Spearman correlations coefficients of POP levels in adipose tissue with serum concentrations (leptin and adiponectin) and gene expression in adipose tissue.** Data represent significant ρ values (*p-value≤0.05; ** p-value≤0.01) M: Men; F: Women.
(PDF)

Table S3 **Spearman correlations of POP levels in serum (ng/g lw) with serum concentrations (leptin and adiponectin) and gene expression in fat tissue.** Data represent significant ρ values (*p-value≤0.05; ** p-value≤0.01)
(PDF)
