# Supplementary material for: Expression of Obesity Markers and Persistent Organic Pollutants Levels in Adipose Tissue of Obese Patients: Reinforcing the Obesogen Hypothesis?
Source: PLoS One. 2014 Jan 10;9(1):e84816. doi: 10.1371/journal.pone.0084816 (PMC3888404; doi:10.1371/journal.pone.0084816)
Supplement: Table S1 — POP concentration levels in SAT/VAT. Data represent the median concentration in ng/g lipid weight and the number of samples below the detection limit. Minimum, maximum concentrations of each compound are shown between brackets. The grey cases indicate compounds that account for at least 50% of the PCB or PBDE burden; these were further analyzed in this paper. SAT = Subcutaneous adipose tissue; VAT = Visceral adipose tissue. (PDF) [file pone.0084816.s001.pdf]

Table S1. **POP concentration levels in SAT/VAT**. Data represent the median concentration in ng/g lipid weight and the number of samples below the detection limit. Minimum, maximum concentrations of each compound are shown between brackets. The grey cases indicate compounds that account for at least 50% of the PCB or PBDE burden; these were further analyzed in this paper. *SAT= Subcutaneous adipose tissue; VAT= Visceral adipose tissue.*

	SAT (n= 50)		VAT (n = 50)	
	Median (min-max)	#samples ≤LOQ	Median (min-max)	#samples ≤LOQ
CB28	0.91 (0.25-27.73)	13	0.99 (0.25-27.22)	3
CB52	1.28 (0.25-8.34)	10	1.09 (0.25-3.93)	7
CB74	5.93 (0.25-58.37)	1	6.02 (1.45-59.16)	0
CB95	≤LOQ (0.25-1.89)	41	≤LOQ (0.25-3.63)	23
CB99	6.02 (0.45-28.82)	0	5.90 (1.60-29.00)	0
CB101	<LOQ (0.25-2.97)	31	0.82 (0.25-5.31)	12
CB105	1.92 (0.54-21.75)	0	1.97 (0.50-21.90)	0
CB118	10.19 (2.26-92.79)	0	10.48 (2.60-90.62)	0
CB128	0.36 (0.02-8.10)	0	0.42 (0.16-7.00)	0
CB138	34.85 (5.02-139.11)	0	34.49 (5.90-141.90)	0
CB146	7.59 (1.22-42.40)	0	7.52 (1.36-42.11)	12
CB149	≤LOQ (0.25-5.40)	35	0.77 (0.25-6.47)	23
CB153	62.59 (8.60-262.65)	0	66.80 (9.15-268.20)	0
CB156	5.89 (0.69-23.96)	0	5.90 (0.74-30.31)	0
CB167	6.93 (0.90-41.62)	0	6.65 (0.98-40.99)	0
CB170	22.06 (2.30-72.09)	0	19.85 (2.35-77.63)	0
CB171	2.00 (0.21-6.93)	0	1.95 (0.26-8.12)	0
CB172	2.30 (0.27-13.93)	0	2.11 (0.29-14.20)	0
CB174	0.42 (0.16-2.38)	0	0.50 (0.20-2.12)	0
CB177	3.20 (0.53-21.26)	0	3.40 (0.59-21.90)	0
CB180	47.75 (4.83-174.49)	0	44.24 (4.90-162.89)	0
CB183	6.08 (0.93-30.38)	0	5.84 (0.94-30.75)	15
CB187	12.05 (1.60-115.05)	0	11.77 (1.64-109.75)	0
CB194	7.24 (0.58-36.00)	0	6.18 (0.59-34.91)	0
CB196_203	7.10 (0.63-41.16)	0	6.50 (0.67-39.91)	0
CB199	3.34 (0.39-40.05)	0	3.16 (0.40-40.84)	0
CB206	1.53 (0.14-8.53)	0	1.44 (0.14-8.41)	0
CB209	1.35 (0.10-7.17)	0	1.26 (0.07-7.54)	0
ΣPCB	276.62 (39.95-1259.97)	0	284.98 (45.20-1232.70)	0
BDE28	0.05 (0.01-0.38)	8	0.04 (0.01-0.44)	6
BDE47	0.48 (0.09-5.40)	0	0.63 (0.12-5.90)	0
BDE99	0.22 (0.05-3.33)	0	0.21 (0.05-2.98)	0
BDE100	0.22 (0.07-1.99)	0	0.21 (0.06-2.26)	0
BDE153	0.92 (0.22-10.67)	0	0.88 (0.26-2.63)	0
BDE154	0.46 (0.01-1.82)	2	0.45 (0.01-1.92)	1
BDE183	0.16 (0.01-2.48)	2	0.13 (0.03-5.87)	0
ΣPBDE	2.70 (0.81-13.66)	0	2.48 (1.05-12.56)	0
