# Supplementary material for: Expression of Obesity Markers and Persistent Organic Pollutants Levels in Adipose Tissue of Obese Patients: Reinforcing the Obesogen Hypothesis?
Source: PLoS One. 2014 Jan 10;9(1):e84816. doi: 10.1371/journal.pone.0084816 (PMC3888404; doi:10.1371/journal.pone.0084816)
Supplement: Table S2 — Spearman correlations coefficients of POP levels in adipose tissue with serum concentrations (leptin and adiponectin) and gene expression in adipose tissue. Data represent significant ρ values (*p-value≤0.05; ** p-value≤0.01) M: Men; F: Women. (PDF) [file pone.0084816.s002.pdf]

Table S2. Spearman correlations coefficients of POP levels in adipose tissue with serum concentrations (leptin and adiponectin) and gene expression in adipose tissue. Data represent significant ρ values (*p-value≤0.05; ** p-value≤0.01) M: Men; F: Women

VAT

	Leptin serum	Leptin M Serum	Leptin F Serum	Adiponectin Serum	Leptin	TNFα	PPARγ	PPARγ T2D	PPARγ NON T2D	Adiponectin
**n**	50	17	33	50	50	50	50	8	42	50
**CB28**				-0.299*	0.290*					
**CB52**				-0.297*						
**CB74**							-0.374**		-0.446**	
**CB99**			-0.402*	-0.316*			-0.313*			-0.304*
**CB105**			-0.383*							
**CB118**							-0.333*			
**CB128**	0.284*									
**CB138**			-0.342*				-0.386**		-0.308*	-0.297*
**CB146**			-0.505**				-0.373**			
**CB153**			-0.381*				-0.387**		-0.331*	
**CB156**			-0.423*		0.340*		-0.352*		-0.324*	
**CB167**			-0.412*		0.280*		-0.330*			
**CB170**			-0.407*		0.330*		-0.334*			
**CB171**			-0.392*				-0.407**		-0.347*	
**CB172**			-0.486**		0.315*		-0.376**		-0.317*	
**CB174**										-0.314*
**CB177**			-0.476**				-0.407**		-0.333*	-0.316*
**CB180**			-0.437*		0.315*		-0.356*	0.714*	-0.306*	
**CB183**			-0.442*				-0.390**		-0.305*	-0.300*
**CB187**			-0.495**				-0.396**		-0.331*	
**CB194**			-0.390*		0.337*		-0.313*	0.714*		
**CB196-203**					0.317*		-0.357*			
**CB199**			-0.435*		0.303*		-0.359*			
**CB206**			-0.387*		0.368**		-0.327*	0.731*		
**CB209**			-0.352*		0.356*		-0.337*			
**ΣPCB**			-0.395*				-0.377**		-0.322*	
**BDE47**						0.359*				
**BDE-99**						0.372**				
**BDE100**					0.321*	0.374**				
**BDE153**	-0.400**		-0.516**		0.335*	0.289*				
**BDE154**			-0.545**	-0.280*	0.318*					
**BDE183**										
**ΣPBDE**	-0.329*		-0.371*		0.286*	0.389**				

SAT

	Leptin serum	Leptin M Serum	Leptin F Serum	Adiponectin Serum	Leptin	Leptin M	Leptin F	TNFα	PPARγ	Adiponectin
**n**	50	17	33	50	50	17	33	50	50	50
**CB28**										
**CB52**										-0.302*
**CB74**										
**CB99**			-0.414*	-0.300*						
**CB105**			-0.401*							
**CB118**			-0.379*							
**CB128**			-0.391*	-0.478**						
**CB138**			-0.463**							
**CB146**			-0.424*							
**CB153**			-0.423*							
**CB156**			-0.469**							
**CB167**			-0.396*							
**CB170**			-0.495**							
**CB171**			-0.448**							
**CB172**			-0.511**							
**CB174**			-0.431*	-0.309*						
**CB177**			-0.525**							
**CB180**			-0.482**							
**CB183**			-0.464**	-0.280*						
**CB187**			-0.524**							
**CB194**	-0.301*		-0.498**							
**CB196-203**	-0.283*		-0.520**							
**CB199**			-0.497**							
**CB206**	-0.282*		-0.505**							
**CB209**			-0.516**							
**ΣPCB**			-0.445**							
**BDE47**							0.379*			
**BDE-99**										
**BDE100**										
**BDE153**	-0.374**		-0.571**							
**BDE154**			-0.551**							
**BDE183**									-0.411**	
**ΣPBDE**			-0.359*							
