# Supplementary material for: Expression of Obesity Markers and Persistent Organic Pollutants Levels in Adipose Tissue of Obese Patients: Reinforcing the Obesogen Hypothesis?
Source: PLoS One. 2014 Jan 10;9(1):e84816. doi: 10.1371/journal.pone.0084816 (PMC3888404; doi:10.1371/journal.pone.0084816)
Supplement: Table S3 — Spearman correlations of POP levels in serum (ng/g lw) with serum concentrations (leptin and adiponectin) and gene expression in fat tissue. Data represent significant ρ values (*p-value≤0.05; ** p-value≤0.01) (PDF) [file pone.0084816.s003.pdf]

Table S3. Spearman correlations of POP levels in serum (ng/g lw) with serum concentrations (leptin and adiponectin) and gene expression in fat tissue. Data represent significant ρ values (*p-value≤0.05; ** p-value≤0.01)

	Serum concentration					VAT expression levels							SAT expression levels					
	Adiponectin	Leptin	Leptin M	Leptin F		Leptin	TNFα	PPARγ	PPARγ T2D	PPARγ NON T2D	Adiponectin		Leptin	Leptin M	Leptin F	TNFα	PPARγ	Adiponectin
**n**	50	50	17	33		50	50	50	8	42	50		50	17	33	50	50	50
**CB138**				-0.366*	**CB138**			-0.457**		-0.400**	-0.286*	**CB138**						
**CB153**				-0.360*	**CB153**			-0.417**		-0.368*	-0.285*	**CB153**						
**CB180**				-0.413*	**CB180**	0.295*		-0.385**		-0.350*		**CB180**						
**ΣPCB**				-0.377*	**ΣPCB**			-0.410**		-0.367*	-0.304*	**ΣPCB**						
**BDE47**					**BDE47**		0.316*					**BDE47**						
**BDE153**		-0.333*		-0.388*														
**ΣPBDE**					**ΣPBDE**		0.359*					**ΣPBDE**						
